# Development and Characterization of Pharmacokinetic Parameters of Fast-Dissolving Films Containing Levocetirizine

**DOI:** 10.3797/scipharm.1205-15

**Published:** 2012-07-22

**Authors:** Dhagla R. Choudhary, Vishnu A. Patel, Usmangani K. Chhalotiya, Harsha V. Patel, Aliasgar J. Kundawala

**Affiliations:** 1 Indukaka Ipcowala College of Pharmacy, New V V Nagar, Anand, Gujarat – 388121, India.; 2 A. R. College of Pharmacy, V V Nagar, Anand, Gujarat – 388120, India.

**Keywords:** Fast dissolving film, Levocetirizine, *In-vitro* dissolution studies, Pharmacokinetics, Drug content

## Abstract

A fast-dissolving film containing levocetirizine, a non-sedative antihistamine drug, was developed using pullulan, xanthan gum, propylene glycol, and tween 80 as the base materials. The drug content of the prepared films was within an acceptable limit as prescribed by the USP. The film exhibited excellent stability for four months when stored at 40 °C and 75% humidity. *In vitro* dissolution studies suggested a rapid disintegration, in which most of levocetirizine (93.54 ± 3.9%) dissolved within 90 seconds after insertion into the medium. Subsequently, Sprague–Dawley rats were used to compare the pharmacokinetic properties of the film preparation administered to the oral cavity, to those with oral administration of the pure drug solution. The pharmacokinetic parameters were similar between the two groups in which AUC_0–t_ (ng h/ml), AUC_0–∞_ (ng h/ml) C_max_ (ng/ml), T_max_ (min), K_el_ (h^−1^), and t_1/2_ (h) of the reference were 452.033 ± 43.68, 465.78 ± 48.16, 237.16 ± 19.87, 30, 0.453 ± 0.051, and 1.536 ± 0.118, respectively, for the film formulation 447.233 ± 46.24, 458.22 ± 46.74, 233.32 ± 17.19, 30, 0.464 ± 0.060, and 1.496 ± 0.293, respectively. These results suggest that the present levocetirizine containing fast-dissolving film is likely to become one of the choices to treat different allergic conditions.

## Introduction

Some patients, particularly pediatric and geriatric patients, have difficulty in swallowing or chewing solid dosage forms. Many pediatric and geriatric patients are unwilling to take these solid preparations due to fear of choking [1]. The fast-dissolving film is a new drug delivery technique to provide medicine to such patients. Fast-dissolving films have acquired great importance in the pharmaceutical industry due to their unique properties and advantages [2, 3]. They undergo disintegration in the salivary fluids of the oral cavity within a minute, where they release the active pharmaceutical ingredient. Their characteristic benefits in terms of patient compliance, rapid onset of action, increased bioavailability (by sometimes bypassing the first-pass effect), and good stability make these films popular as the dosage form of choice [4–6].

An allergy is a hypersensitive disorder of the immune system. Allergic reactions occur due to environmental substances known as allergens. An allergy is one of four forms of hypersensitivity and is called type I hypersensitivity. It is characterized by the excessive activation of certain white blood cells called mast cells and basophils by a type of antibody known as IgE, resulting in an extreme inflammatory response. Mild allergies like hay fever are highly prevalent in the human population and cause symptoms such as allergic conjunctivitis, itchiness, and runny nose. A variety of tests now exist to diagnose allergic conditions; these include testing the skin for responses to known allergens or analyzing the blood for the presence and levels of allergen-specific IgE. Treatments for allergies include allergen avoidance, use of antihistamines, steroids or other oral medications, immunotherapy to desensitize the response to the allergen, and targeted therapy [7–9].

Levocetirizine (LCZ) is a third-generation non-sedative antihistamine, which works by blocking histamine receptors. It does not prevent the actual release of histamine from mast cells, but prevents it binding to its receptors. LCZ prevents the release of other allergic chemicals produced in response to allergens and the increased blood supply to the area, and provides relief from the typical symptoms of hay fever [10].

Levocetirizine is bitter in taste. The bitterness of the drug was lessened by using hydroxyl propyl β-cyclodextrin complexes as reported by Mahesh in 2010 and Dinge in 2008 [11, 12].

Pullulan is a natural polysaccharide produced from starch by cultivating the black yeast, *Aureobasidium pullulans*. It is a white, tasteless, odourless, and water-soluble powder. Pullulan (PI-20 grade) is the deionised form of pullulan, having an average molecular weight of 200,000 daltons and possesses excellent film-forming properties [13, 14].

## Materials and Methods

### Materials

Levocetirizine dihydrochloride (LCZ) was obtained as a gift sample from Cipla Ltd., Ahmedabad, Gujarat, India. Neotame, hydroxyl propyl β-cyclodextrin (HPβ-CD), and xanthan gum were provided by Alkem pharmaceutical Pvt Ltd. Mumbai, India. Pullulan gum was provided as a gift sample by Gengwal Chemicals, Mumbai, India. Propylene glycol (PG) and tween 80 were purchased from Sigma chemicals.

### Preparation of fast-dissolving films

The basic components of FDFs were LCZ : HPβ-CD complex (0.432% w/v, 1:1 molar ratio), pullulan (2% w/v), xanthan gum (0.4% w/v), propylene glycol (0.2% w/w), and tween 80 (0.1% w/w) in 25 ml of water. All of these excipients were dissolved in water by using a magnetic stirrer to obtain a homogenous dispersion. Then it was allowed to be kept aside for 8 h to remove air bubbles, and then 25 ml of the dispersion was cast onto a glass petridish (Borosil, Gujarat, India) having a surface area of 64 cm^2^ and a 1.3 cm wall height. The dispersion was dried in a hot air oven at 45–50 °C. After drying, the films were removed with the help of a sharp blade and kept in a desiccator for 24 hrs before cutting it into small pieces, having an area of 6 cm^2^ for each film. Films with air bubbles, cuts, or imperfections were excluded from further study. Selected films were subjected to different evaluation parameters [15, 12].

### Appearance, weight, tensile strength, disintegration time, and content uniformity

The prepared films were evaluated for different parameters like: appearance, weight [15], tensile strength [16, 17], content uniformity [11], and disintegration time [18].

### In vitro dissolution studies

An *in vitro* dissolution study was performed for selected films for three minutes in the USP paddle apparatus using a pH 6.4 buffer solution. The dissolution medium was kept at 37 ± 0.5 °C and rotated at 500 rpm. The samples (5 ml) were withdrawn after every 30 sec interval and replaced with a fresh buffer (pH 6.4) solution. One ml of the sample was then taken and diluted up to 10 ml in a volumetric flask. The samples were analyzed for the drug content using a UV spectrophotometer at 231 nm. Dissolution was performed three times for each formulation to calculate the drug-release profile [12].

### Stability study

Short-term stability studies were performed for optimized films, and were placed in plastic containers and exposed to 40 ± 0.5 C and 75± 5% RH (ICH guidelines) for a period of four months. Different film properties like physical appearance of the film, mechanical properties, and drug content were evaluated at intervals of one week [19].

### Determination of pharmacokinetic parameters

In this study, Sprague–Dawley rats were used. Their mean weight was 264.66 ± 8.96 g in the range of 250–275 g. Animals were housed in a room maintained on a 12-h light/dark cycle at 23 ± 2 °C with free access to food and water. The experimental procedures were approved by the Committee for the Care and Use of Laboratory Animals at the IICP (Protocol number: IICP/PH/12-2010/02). For the administration of sample (film) preparation, 50 μl aliquot of distilled water was dropped into the rat oral cavity under light ether anesthesia, then two halves (1 cm×0.5 cm) of the film preparation were applied to the buccal cavity bilaterally. For oral administration, rats were given 1mg/kg of the LCZ dose (equivalent to the body weight of rat) in solution containing 1 ml water, under light ether anesthesia [20]. Blood specimens were taken (every 0.5 ml) in a centrifuge plastic capillary tube by the intraorbital route at 0 min, 30 min, 1 h, 2 h, 4 h, 6h, and 12 h after drug administration. Blood was subjected to centrifugation at 10,000 rpm for 15 min, then plasma was taken in a polyethylene tube and stored at −20 °C until its assay. The concentration of levocetirizine was determined by HPLC-UV at 230 nm [21]. The HPLC-UV system that was used consisted of a pump (Perkin Elmer, USA) with universal loop injector (Rheodyne) of injection capacity 20 μl. The detector consisted of a UV detector; the reversed-phase column that was used was RP-C_18_ (5μm size, 250 mm 4.6 mm i.d.) at ambient temperature. The mobile phase was a mixture of Acetonitrile : Buffer (KH_2_PO_4_, pH 5.5, 0.02 M, pH was adjusted with NaOH) 70:30% v/v [22, 23].

### Pharmacokinetic parameters and statistical calculation

Pharmacokinetic parameters were derived from the plasma concentration vs. time plot. The peak plasma concentration (C_max_) and the time to attain peak concentration (T_max_) were obtained from experimental points of the plot. The other pharmacokinetic parameters were determined by using Kinetica software (Version 5, Adept Scientific, UK). The data of the reference (oral solution of the pure drug) and sample (film) were compared and statistically evaluated by Student’s t-test.

## Results and Discussion

### Appearance, weight, tensile strength, disintegration time, and content uniformity

Prepared films were opaque in appearance. The weight and tensile strength of the films were found to be 83 ± 6.9 mg and 6.18 ± 1.45 N/mm^2^, respectively. The drug content was found to be 96.4 ± 4.5%, and the results suggested that the drug content was within the prescribed limit (85–115%) as specified by the USP. Films were disintegrated in 16 ± 1.56 sec.

### In vitro dissolution studies

An *in vitro* dissolution study was performed for the films for three minutes in a USP paddle apparatus using a pH 6.4 buffer solution. The prepared films released their 93.54 ± 3.9% of drug in 90 sec.

### Stability study

A stability study of selected films at 40 ± 2 °C/75% RH ± 5% RH was carried out for four months. The results are shown in Table 1. The result of the stability study revealed that there was no significant difference in the drug content of the formulation. The formulations showed satisfactory tensile strength at 40 °C and 75% RH. The physical appearance did not change considerably. These finding suggest that films are stable at high temperatures.

### Determination of pharmacokinetic parameters for the reference (oral solution of the pure drug) and sample (film)

#### Validation of method

The intra-assay and inter-assay precision were determined with a quality control sample spiked with three different concentrations of levocetirizine (50, 200, and 400 ng/ml) as shown in Table 2. The intra-assay CV for the three quality control samples ranged from 12 to 15% and the inter-assay CV ranged from 8 to 17%, where the acceptable value is less than or equal to 20% for the CV as proposed by the FDA. High accuracy and precision were achieved by the external standard method of calibration, thus an internal standard was not required [24].

#### Comparison of concentrations of drug in plasma at different time intervals for the reference (oral solution of the pure drug) and sample (film)

**Fig. 1:**
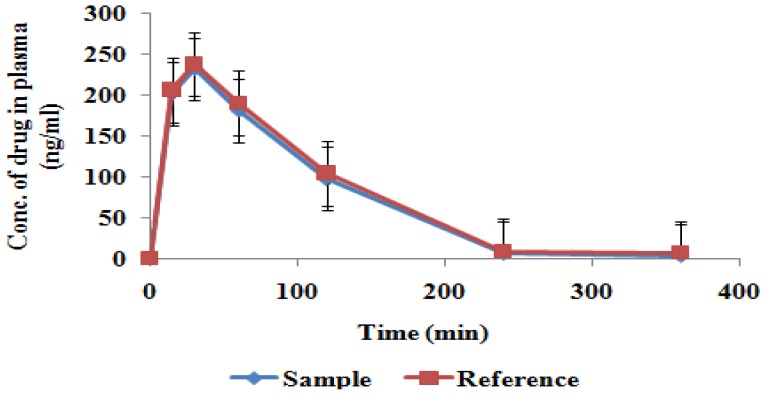
Comparison of the concentration of levocetirizine in plasma for reference (oral solution of the pure drug, n=6) and sample (film); (mean ± SD, n = 6).

As reported earlier, the HPLC determination of levocetirizine in human plasma was assayed by Macek in 1999 [24]. In our study, we have successively measured the concentration of levocetirizine dihydrochloride in rat plasma by using HPLC with UV detection. Table 3 and Figure 1 show the time course of changes in levocetrizine concentrations in rat plasma, after oral administration of levocetirizine solution or administration of fast-dissolving film to the oral cavity. The pattern of changes in plasma concentrations was similar between the two groups, although the concentrations were slightly, but not significantly, higher in the oral solution-treated group.

Since the film and solution remain in the oral cavity for a few seconds (5–10 sec), there are fewer chances of pre-gastric absorption, so that this study was carried out only on blood profiles.

### Comparison of pharmacokinetic parameters of levocetirizine between reference (oral solution of the pure drug) and sample (film) in rats

The pharmacokinetic parameters were evaluated from the profiles of 12 rats (six rats each for the reference and sample). The pharmacokinetic parameters for the reference (oral solution of the pure drug) were found as follows: AUC_0–t_ 452.033 ± 43.68 ng h/ml, AUC_0–∞_ 465.78 ± 48.16 ng h/ml, C_max_ 237.16 ± 19.87 ng/ml, T_max_ 30 minutes, K_el_ 0.453 ± 0.0519 h^−1^, and t_1/2_ 1.536 ± 0.118 h. The pharmacokinetic parameters for the sample (film) were found as follows- AUC_0–t_ 447.233 ± 46.24 ng h/ml, AUC_0–∞_ 458.22 ± 46.74 ng h/ml, C_max_ 233.32 ± 17.198 ng/ml, T_max_ 30 minutes, K_el_ 0.464 ± 0.0601 h^−1^, and t_1/2_ 1.496 ± 0.293 h (Table 4).

The Analysis of Variance (ANOVA) for data from AUC_0–t_, AUC_0–∞_, C_max,_ and T_max_ showed no statistically significant difference between the two formulations. This suggests that both of the formulations have similar blood profiles. The respective 95% CI of the ondansetron sample/reference percent ratios were 98.93% (91.14–118.36%) for AUC_0–t_*,* 97.39% (92.34–115.61) for AUC_0–∞_, and 98.38% (89.34–114.41%) for C_max_, while the acceptable range was 80–125% for AUC_0–t_ and AUC_0–∞_, and 70–143% for C _max_ as proposed by the FDA and the ANVISA (Table 5).

The results of this study showed that there was no significant difference in the blood profile of the reference (oral solution of the pure drug) and the sample (film) as shown in Table 4.

## Conclusion

The prepared films of levocetirizine revealed excellent mechanical properties, drug content, and stability. The pattern of changes in plasma concentrations of the drug was similar between the two groups, although the concentrations were slightly, but not significantly, higher in the oral solution-treated group. No significant differences were observed in the pharmacokinetic parameters obtained from rats with the oral administration of levocetirizine solution of the pure drug and those with the film. Hence, the present fast-dissolving films containing levocetirizine are considered to be potentially useful for treating different allergic conditions.

Based on the results, it is concluded that FDFs are convenient and reliable dosage forms that can get rid of the common problems associated with solid dosage forms.

## Figures and Tables

**Tab. 1:** Evaluation of different parameters during stability studies

Time (Month)	Parameters		

	Appearance	Tensile strength (N/mm^2^)	Drug content (%)
1	+++	6.11 ± 1.65	96.34 ± 2.09
2	+++	5.98 ± 1.59	96.62 ± 1.69
3	+++	5.85 ± 1.66	95.78 ± 1.67
4	++	5.81 ± 1.17	94.74 ± 1.92

+++…flexible and opaque film, crystallization of drug not observed;

++…flexible and opaque film, crystallization of drug observed.

**Tab. 2:** Intra-assay and inter-assay variation for reference (oral solution of the pure drug) and sample (film); (mean ± SD, n = 3).

QC sample	Levocetirizine added (ng/ml)	Intra-Day		Inter-Day	
	
		Area (mean ± SD)	CV (%)	Area (mean ± SD)	CV (%)
LQC	50	15473 ± 2471	15.9	14233 ± 2486	17.4
MQC	200	54021 ± 6704	12.4	55660 ± 4646	8.3
HQC	400	91115 ± 11939	13.1	88248 ± 8997	10.1

QC = Quality Control; LQC = Low Quality Control; MQC=Medium Quality Control; HQC= High Quality Control; CV = Coefficient of variance.

**Tab. 3:** Plasma levels of levocetirizine in plasma for reference (oral solution of the pure drug) and sample (film); (mean ± SD, n = 6).

Time (min)	Levocetirizine content (ng/ml)	

	Oral solution (reference)	Film (sample)
0	0	0
15	207 ± 12	205 ± 9
30	237 ± 19	233 ± 17
60	187 ± 17	184 ± 12
120	101 ± 9	100 ± 9
240	8 ± 2	7 ± 2
360	6 ± 2	5 ± 2

**Tab. 4:** Comparison of pharmacokinetic parameters of levocetirizine between the sample (film) and reference (oral solution of the pure drug) in Sprague-Dawley rats (mean ± SD, n = 6).

Parameters	Reference (Oral solution)	Sample (Film)
AUC_0–t_ (ng. h/ml)	452.033 ± 43.68	447.233 ± 46.24
AUC_0–∞_ (ng. h/ml)	465.78 ± 48.16	458.22 ± 46.74
C_max_ (ng/ml)	237.16 ± 19.87	233.32 ± 17.19
T_max_ (minute)	30	30
K_el_ (h^−1^)	0.453 ± 0.519	0.464 ± 0.601
t_1/2_ (h)	1.536 ± 0.118	1.496 ± 0.293

**Tab. 5:** Sample (film) to reference (oral solution of the pure drug) ratio of different pharmacokinetic parameters for levocetirizine (mean ± SD, n = 6).

Pharmacokinetic parameters	Sample (Film)	References (Oral solution)	Sample/Reference ratio (%)
AUC_0–t_ (ng. h/ml)	447.233	452.033	98.93
AUC_0–∞_ (ng. h/ml)	458.220	465.780	97.39
C_max_ (ng/ml)	233.32	237.16	98.38
